# Modularity-based credible prediction of disease genes and detection of disease subtypes on the phenotype-gene heterogeneous network

**DOI:** 10.1186/1752-0509-5-79

**Published:** 2011-05-20

**Authors:** Xin Yao, Han Hao, Yanda Li, Shao Li

**Affiliations:** 1 MOE Key Laboratory of Bioinformatics and Bioinformatics Division, Tsinghua National Laboratory for Information Science and Technology, Tsinghua University, Beijing 100084, China

## Abstract

**Background:**

Protein-protein interaction networks and phenotype similarity information have been synthesized together to discover novel disease-causing genes. Genetic or phenotypic similarities are manifested as certain modularity properties in a phenotype-gene heterogeneous network consisting of the phenotype-phenotype similarity network, protein-protein interaction network and gene-disease association network. However, the quantitative analysis of modularity in the heterogeneous network and its influence on disease-gene discovery are still unaddressed. Furthermore, the genetic correspondence of the disease subtypes can be identified by marking the genes and phenotypes in the phenotype-gene network. We present a novel network inference method to measure the network modularity, and in particular to suggest the subtypes of diseases based on the heterogeneous network.

**Results:**

Based on a measure which is introduced to evaluate the closeness between two nodes in the phenotype-gene heterogeneous network, we developed a Hitting-Time-based method, CIPHER-HIT, for assessing the modularity of disease gene predictions and credibly prioritizing disease-causing genes, and then identifying the genetic modules corresponding to potential subtypes of the queried phenotype. The CIPHER-HIT is free to rely on any preset parameters. We found that when taking into account the modularity levels, the CIPHER-HIT method can significantly improve the performance of disease gene predictions, which demonstrates modularity is one of the key features for credible inference of disease genes on the phenotype-gene heterogeneous network. By applying the CIPHER-HIT to the subtype analysis of Breast cancer, we found that the prioritized genes can be divided into two sub-modules, one contains the members of the Fanconi anemia gene family, and the other contains a reported protein complex MRE11/RAD50/NBN.

**Conclusions:**

The phenotype-gene heterogeneous network contains abundant information for not only disease genes discovery but also disease subtypes detection. The CIPHER-HIT method presented here is effective for network inference, particularly on credible prediction of disease genes and the subtype analysis of diseases, for example Breast cancer. This method provides a promising way to analyze heterogeneous biological networks, both globally and locally.

## Background

Disease gene prediction is one of the most important aims in biological and medical sciences. Network-based evidence as well as inference approaches has become more and more attractive in the research field of disease-causing gene discovery, and a variety of methods have been developed recently from this point of view [1-5]. Researchers also attach great importance to special features embedded in biological networks especially the protein-protein interaction (PPI) network for deeply understanding molecular mechanism of common human diseases [6-15]. Since genetic diseases are genetically or phenotypically similar, it is promising to combine the protein-protein interactions and the phenotype similarities to a phenotype-gene heterogeneous network to infer the candidate disease genes [1-4]. The so-called "phenotype-gene heterogeneous network" reflects a holistic view of complex relationships among various phenotypes and phenotypes, phenotypes and genes, as well as genes and genes, which consists of the phenotype-phenotype similarity network, gene-disease association network and protein-protein interaction network, respectively. Based on such a heterogeneous network, we propose a regression model named CIPHER (Correlating protein Interaction network and PHEnotype network to pRedict disease genes) to quantify the concordance between candidate genes and target phenotypes [2]. The algorithm of random walk is also proposed to prioritize the candidate disease genes in protein-protein interaction networks [3] and then a random walk with restarts (RWR) method is extended to the above heterogeneous network [4].

In general, the network-based disease-gene discovery methods make use of information from both the topological structure and the associations between diseases and genes. The basic assumption is that similar disease phenotypes are caused by functionally related genes and these genes are likely to be close to each other on the protein-protein interaction networks, so that network modules are formed [15-18,5]. Here the network module in computation refers to a group of genes exhibiting network proximity, and in biology refers to certain functional units such as protein complexes, signaling or metabolic pathways and transcriptional programs [16-19,5]. Therefore, the algorithms in [3] prioritize candidate genes based on their closeness to known disease genes. After the similarity information between the phenotypes is provided by van Driel *et al. *through text mining technology [17], the phenotype similarity and the protein-protein interactions are combined together for the prioritization of the candidate disease genes [1,2,4].

However, so far little modularity analysis on the phenotype-gene heterogeneous networks has been done. The predicted results from the network inference methods need to be tested to see whether they form the modules and to which corresponding biological function they are related. In this paper, the network inference methods are further developed to measure the modularity property of the disease-gene prediction results. Furthermore, we also provide the method to infer the relationship between the subtypes of diseases and the modules formed by these predicted results.

### Inference on the phenotype-gene heterogeneous network

For the network-based inference, a candidate gene *g *is prioritized to be a potential disease-causing gene of the target phenotype *p *if one or both of the followings are satisfied:

1. The gene *g *is close to some disease-causing genes associated with *p*.

2. The gene *g *is close to some phenotypes which are highly similar to *p*.

Hence one key point is to define the closeness between two nodes in the network, and this will be used to measure the similarity between the nodes based on the network topology [1,2]. Currently the nearest-neighbor method considers the direct interactions information and ignores the long-range interactions. The shortest-path method considers the length of the shortest path connecting two nodes but ignores the number of short paths between them. The random walk with restart method [3,4] combines the local and global network information to enhance the prediction performance.

Another key point is the priori information known about each target phenotype, the known disease-genes and the similar phenotypes. In the phenotype-gene heterogeneous network, for each given phenotype *p*, its known causing genes and the similar phenotypes are represented as the nodes which link to *p *directly, and these nodes are termed as the adjacent nodes of the target phenotype *p *in the heterogeneous network. The paths between *p *and any other nodes have to cross this adjacency set. Therefore, the prioritization can be carried out by measuring the closeness between the candidate genes (namely all genes in the protein-protein interaction network) and these adjacent nodes.

**Figure 1:**
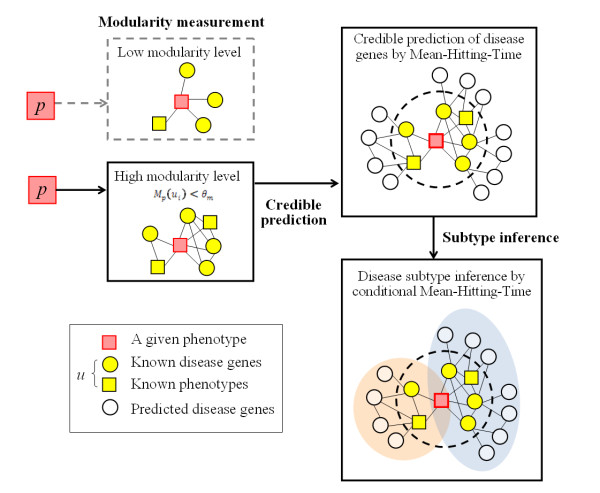
**The flowchart of the CIPHER-HIT method**. In the CIPHER-HIT method, we first evaluate the modularity of the adjacent nodes around the given phenotype, and then select credible reference for disease gene prediction by the Mean-Hitting-Time, which will be further subjected to detection of disease subtypes by the conditional Mean-Hitting-Time on the phenotype-gene heterogeneous network.

In this paper, we introduced a closeness measure based on the methods of Mean-Hitting-Time and conditional Mean-Hitting-Time, which not only capture the global relationships within the phenotype-gene heterogeneous network, but also free to rely on any priori parameters. Moreover, by studying the different relationships to different adjacent nodes, we assume that the prioritized genes can be further divided into sub-modules which may correspond to the subtypes of the disease. And the conditional Mean-Hitting-Time can be applied to discover such disease subtypes. The present Hitting-Time-based method with the flowchart illustrated in Figure 1 is called CIPHER-HIT, as a continuation of our CIPHER method [2].

### Candidate disease genes prioritization: which are the most credible?

**Figure 2:**
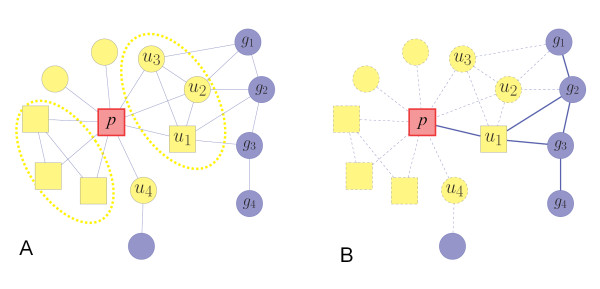
**Illustration of the network inference and modularity measure in CIPHER-HIT**. The circle nodes represent the genes and the rectangle nodes represent the phenotypes. The red node denotes the target phenotype *p*. The yellow nodes (*u*) denote the adjacent nodes of *p*, i.e. the set , referring to either genes or phenotypes. **(A) **The dashed ellipses enclose the adjacent nodes which share high topological similarity. The nodes *u*_1_, *u*_2 _and *u*_3 _are close to each other. Therefore candidate nodes *g*_1_, *g*_2_, *g*_3 _and *g*_4_, which can more easily form a module in the protein-protein network, will be prioritized as the potential disease-genes. The group *u*_1_, *u*_2 _*u*_3_, *g*_1_, *g*_2_, *g*_3 _and *g*_4 _will be inferred as a module related to phenotype *p*. **(B) **The illustration of the meaning of the conditional Mean-Hitting-Time . Among the paths from the candidate genes *g *to the phenotype *p*, the influence of the paths passing the adjacent nodes other than *u*_1 _are excluded, which are illustrated as dashed lines.

Based on the closeness measure of the phenotype-gene heterogeneous network, the candidate genes can be prioritized according to their topological similarities of the adjacent nodes. The inference is of the same spirit as the methods in [1-4]. However, some disease-causing genes are likely to be topologically similar, whereas some others will be dispersed among the heterogeneous network. As shown in Figure 1, for a phenotype that has many known disease genes and similar phenotypes, we probe the relationships among these adjacent nodes and suppose that if an adjacent node (a known disease gene or a similar phenotype to the target phenotype) has higher topological similarity with the others, then it will be a more credible reference gene or phenotype for inference of disease-causing genes. Here the topological similarity between two nodes means their closeness or connectivity strength on the network, which can be defined as the Mean-Hitting-Time of the random walk. We consider this hypothesis is reasonable since it is widely assumed that similar phenotypes may be caused by functionally close related genes [15,16], thus if more information about protein-protein interactions, gene-phenotype associations as well as phenotype-phenotype similarities is known, higher inference accuracy in gene-phenotype relationship inference will be achieved. As a graphic illustration shown in Figure 2, nodes *u*_1_, *u*_2 _and *u*_3 _will be the more credible references than *u*_4 _since they are close to each other. Therefore, our CIPHER-HIT method developed is firstly used to measure the connectivity strength between one adjacent node and the others, and then those candidate genes near the credible reference will be marked as the ones being more likely to form modules in the network.

### Gene sets inference for the disease subtypes

Identifying subtypes of diseases such as cancer is of critical importance for predicting clinical outcomes as well as designing more-specific therapies for patients, facilitating a new era of translational medicine and personalized medicine [20,21]. The intrinsic cancer subtypes have been studied in different ways by using histology, molecular pathology, genetic mutation and gene-expression information [21]. The classification of human cancer has become more and more informative as the detailed molecular analysis is provided. For example, the molecular heterogeneity in tumor can be recognized according to the different patterns of the gene expression information [20-22]. Interestingly, Li *et al. *recently reported an integrative network analysis method to identify recurrent network modules that contribute to Breast cancer metastasis by using a set of tumour gene microarrays [23]. Since molecular network modules have been detected in cancer subtypes [23], it is possible to use network modules to further classify Breast cancer into subtypes.

It is well accepted that similar phenotypes may be caused by functionally close related genes [1-16]. An extension of this assumption would be that genes related to different subtypes are likely to form distinct protein-protein interaction modules, which is a common indicator of gene functional relationship [24].

Thus, our CIPHER-HIT method is further used to identify the sub-groups of genes corresponding to the cancer subtypes. Such groups of genes are called sub-modules in the network, and the main task of our method is to identify the gene sets related to different subtypes of a target disease (or phenotype). In cases where the heterogeneity information of a phenotype is included in its adjacent nodes, it is promising to further classify the prioritized genes based on such information. The similar phenotypes and their associated genes have also provided information for identifying the sub-modules. For example, the phenotype node representing FANCONI ANIMIA has high topological similarity to the phenotype node BREAST CANCER. Recent studies demonstrate that genes FANCA, FANCB, FANCC, FANCD2, FANCE, FANCF and FANCG associated with Fanconi animia are closely related to the susceptibility of Breast cancer [25,26]. These genes can be prioritized to be associated with Breast cancer by CIPHER-HIT successfully. In addition, by discriminating the adjacent nodes through which these genes are prioritized, they can also be marked as the sub-module corresponding to the subtype of Fanconi animia related Breast cancer.

Thus, in this work, we develop a method to reveal the relationship between each prioritized gene and each adjacent node so that the hierarchical clustering method is applied to discover the potential subtypes of the target phenotype. These results are meaningful for further biomedical and experimental researches, since they help to focus on the genes which are likely to form the sub-modules corresponding to the potential subtypes of diseases.

## Results and Discussion

### CIPHER-HIT: the topological closeness measure based on the Mean-Hitting-Time

The CIPHER method [2] and the random walk with restart method (RWR) [3,4] are the approaches which reflect the global structural information of the phenotype-gene heterogeneous network, while the parameters such as the restart rate in RWR, which are related to the performance, are required to be pre-set. In the CIPHER-HIT method, we present a new closeness measure between two nodes based on the Mean-Hitting-Time of the random walk on the heterogeneous network. Although this measure is developed from the same mathematical background as the random walk with restart method [3,4], it both reflects the global topological information very well and refrains from setting up a difficult-to-explain priori parameter. Moreover, one extension of this measure - the conditional Mean-Hitting-Time can be used to discover modularity characteristics on the phenotype-gene heterogeneous network and contribute to disease subtype inference.

For a random walk on the network, the Hitting-Time to the set of nodes *B*, denoted by *τ_B_*, is defined as the first time when *B *is visited. The Mean-Hitting-Time of a random walk starting from the node *a *to the set *B *is defined as(1)

where ℙ*_a_*(*τ_B _*= *k*) refers to the possibility that a random walk starting form node *a *hits the set *B *at a time point *k*, and *k *is the summing target ranging from 1 to positive infinite.

The Mean-Hitting-Time include all the router information between the node *a *and set *B*. We define the closeness measure between node *a *and set *B *by the scaled Mean-Hitting-Time (MHT) with the maximal value for all nodes *a*' on the network,(2)

Here  can be inconveniently large in actual calculation, so we scale it to ensure the range of MHT is between 0 and 1.

Furthermore, if we need a topological closeness between the node *a *and the set *B *without the influence of a given set of nodes, *A*, the conditional Mean-Hitting-Time will be a natural choice. It is defined as(3)

where ℙ*_a_*(*τ_B _*= *k*|*τ_B _*<*τ_A_*) refers to the possibility that a random walk starting form node *a *hits the set *B *at a time point *k*, conditioning on the same random walk hits the set *B *before it hits the set *A*.

Similarly, we define the scaled conditional Mean-Hitting-Time (CMHT) CMHT(*a*, *B*|*A*), as the closeness measure between node *a *and set *B*, without the influence of set *A*,(4)

We also scale CMHT to the range between 0 and 1 to avoid the inconvenient large  in actual calculation. Both of the closeness measures defined in Equation (2) and Equation (4) can be computed explicitly without any preset parameters (see detailed computational methods in Material and Methods).

### Performance of CIPHER-HIT in credibly predicting diseases-causing genes

In this work, we firstly apply the scaled Mean-Hitting-Time in ranking candidate disease-causing genes based on the phenotype-gene heterogeneous network. The adjacency set of a certain node *n *on the network is defined as all those nodes linked to *n *by an edge on the network, either a 1-valued association as in the protein-protein interaction network and gene-disease association network, or a positively weighed association as in the phenotype-phenotype similarity network filtered by a threshold (see Material and Methods). For each given phenotype *p *having an adjacency set , we compute MHT(*g*,{*p*}) for each candidate gene *g*. After ranking MHT(*g*,{*p*}) from the smallest to the largest, a gene *g *will be prioritized as the potentially causal gene associated with phenotype *p *if MHT(*g*,{*p*}) <*θ_R_*, where *θ_R _*is the filtering threshold. The detailed setting of *θ_R _*will be discussed at middle of the second to last paragraph of this subsection. The ranking information of each gene *g *is recorded as the ranking position RANK*_p_*(*g*). For the target phenotypes *p *which have many nodes in the adjacency set , we introduce the Modularity Level through conditional Mean-Hitting-Time as below:(5)

which can be used to test the connectivity strength between *u_i _*and other adjacency nodes. Note that a smaller value of the conditional Mean-Hitting-Time (*M_p_*(*u_i_*)) indicates a higher modularity level, namely a stronger connection between the adjacent node (*u_i_*) and other nodes in the adjacency set . By calculating the minimum conditional Mean-Hitting-Time, we assess the modularity level of one node *p *on the network with regard to its adjacency node *u_i _*as the maximum connectivity strength between other adjacency nodes *u *and *u_i_*. Different from the concept of topological similarity between two nodes, the modularity level of one node with regard to another takes the other adjacency nodes into consideration, and serves as the measure of connectivity strength among more than two connected nodes. Then we set a threshold *θ_M _*to distinguish the adjacent nodes so that  which satisfies *M_p_*(*u_i_*) <*θ_M _*will be marked as the one with high connectivity strength to the other adjacent nodes.

Hence the adjacent nodes are divided into two parts,  and  which are defined as(6)(7)

According to the definition above,  denotes the adjacent nodes *u *including disease-genes associated with *p *or phenotypes similar to *p *that are strongly connected with each other. For any , the random walk starting from *u_i _*will reach *u_j _*easily without passing *p*. This feature is illustrated in Figure 2B.

Next, we analyze the prioritized genes for target phenotype *p*. We measure the closeness between each gene to the nodes in  without the influence of the nodes in . We compute  for each gene *g *and then rank results from the smallest to the largest, so that we record the ranking position r'*_p_*(*g*). By comparison of RANK*_p_*(*g*) and RANK'*_p_*(*g*) for each prioritized genes, and if RANK*_p_*(*g*)/RANK'*_p_*(*g*) > 1, we conclude that gene *g *is in association with the node *p *because it is close to the adjacent nodes in set , and these genes are marked as the most credible predicted results.

**Figure 3:**
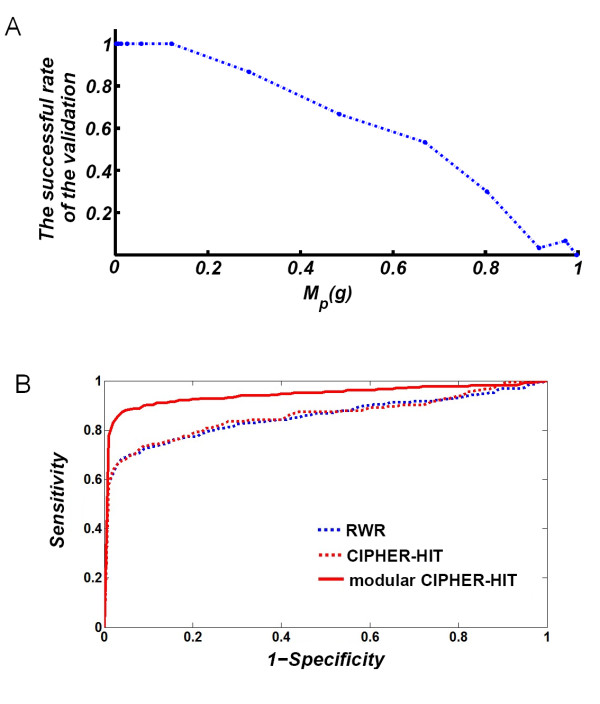
**Results of the genome-wide cross-validation for disease gene prioritization**. **(A) **The conditional Mean-Hitting-Time (*M_p_*(*g*)) is calculated by Equation (5). Results showed that genes with high modularity levels to the other adjacent nodes with small *M_p_*(*g*) values will be more likely to be successfully prioritized during the validation. **(B) **The receiver operating characteristic (ROC) curves of the genome-wide leave-one-out cross-validation. The horizontal coordinates (1-Specificity) refer to values of *θ_R_*, while the vertical coordinates (Sensitivity) refer to the true-positive rate corresponding to *θ_R_*. The red solid line denotes the inference of modular CIPHER-HIT based on the nodes in , i.e. only the adjacent node *u *of *M_p_*(*u*) <*θ_M _*= 0.3 are used for inference. The dashed lines both denote the inference based on the nodes in , i.e. only the adjacent node *u *of *M_p_*(*u*) ≥ *θ_M _*= 0.3 are used for inference, where the blue dashed line denotes results from the random walk with restarts (RWR) method, and the red dashed line denotes results from the CIPHER-HIT method.

The performance of CIPHER-HIT is evaluated by a genome-wide leave-one-out cross-validation. The candidate gene set is defined as all genes on the heterogeneous network. The set of validated genes are the known associated genes of the disease phenotypes. At each round of the validation, one gene associated with the target phenotype will be chosen as a validated sample, the link between the chosen gene-node and the phenotype-node is removed and the scaled Mean-Hitting-Time from each gene-node to the target phenotype-node (the one from which a link is removed) is re-computed and ranked from the smallest to the largest. Note that a disease gene can be associated with many phenotypes. Therefore, the gene is deemed to come from different samples when the validation is carried out for different phenotypes. If a sample for validation satisfies MHT(*g*,{*p*}) <*θ_R_*, it will be considered a successful prediction. The results of the leave-one-out cross validation are shown as the receiver operating characteristic (ROC) curves in Figure 3, where the horizontal coordinates (1-Specificity) refer to values of *θ_R_*, and the vertical coordinates (Sensitivity) refer to the true-positive rate corresponding to *θ_R_*. The validation on the disease genes in the set  produces obviously poorer performance than the validation on the disease genes in the set . This is reasonable since the genes in  are likely to be close to the other known disease genes or phenotypes similar to *p*. From the results shown in Figure 3A, we found that the higher the modularity level a gene to the other adjacent nodes is, the higher the successful rate of the validation will be. When compared with the random walk with restarts (RWR) method [4], we found that the ROC curves of both RWR and CIPHER-HIT are comparable. However, when taking into account the modularity levels, only the adjacent node *u *of *M_p_*(*u*) <*θ_M _*= 0.3 are used for inference in CIPHER-HIT method, the so-called modular CIPHER-HIT can significantly improve the performance of disease gene predictions, making it possible to reach the credible prediction of disease genes (Figure 3B).

Note that though we mark the prioritized genes that are close to the adjacent node in , we do not exclude the other prioritized genes. The nodes in  are also available to form modules with other genes but they might not be exhibited because of the incompleteness of the network information. Since the genes in  already exhibit the inclination to have tight relationship, we suggest the marked genes be selected for further biological investigation with high priority.

### Disease subtype inference by CIPHER-HIT

The development of a reliable method to identify disease subtypes will not only enhance our understanding of disease mechanism, but also provide principles for designing a tailored diagnosis and treatment for patients. For a long time, identification of disease subtypes by phenotype associations of patients is of highly importance for assigning individual treatments in the medical community, especially in traditional Chinese medicine which holds "*Bian-ZHENG-Lun-Zhi*" (Syndrome differentiation and treatment for disease) as its core concept [27]. Inspired by such a rationale [27], we further note that in the heterogeneous networks, the adjacent set of a target phenotype can be used not only to predict potential disease-causing genes, but also to reveal further structural relationships among the genes with regard to their contributions to disease phenotypes. If the prioritized genes of a query phenotype can be further grouped into several classes according to different functions, then the sub-modules in the network are expected to be distinguished to correspond to these sub-groups of genes.

Thus, in the framework of CIPHER-HIT, given a queried phenotype *p*, suppose its adjacent node and prioritized gene set are {*u*_1_, ···, *u_m_*} and {*g*_1_, ···, *g_k_*}, respectively, then we define(8)

which measures the closeness between the gene *g *and the adjacent node *u_i _*without the influence of the other adjacent nodes. Note that the selection of prioritized genes set {*g*_1_, ···, *g_k_*} here is addressed by fitting a threshold *θ_R _*in the step of disease gene prioritization. Since we filter credible disease gene set by the Mean-Hitting-Time MHT(*g*, {*p*}), we naturally choose the threshold as the critical point of the empirical distribution function of MHT(*g*, {*p*}) for all genes on the network (See case study for Breast cancer). Then, as shown in Figure 2B, the value c*_p_*(*g*, *u*_1_) will only depend on the path connecting gene *g *and *p *trough the adjacent node *u*_1_, without considering the paths passing other adjacent nodes *u*_2_, *u*_3_, ···. After computing c*_p_*(*g*, *u_i_*) for all the adjacent nodes of *p*, we can get feature vectors of the prioritized genes *g*. By the alignment of such feature vectors of all the prioritized genes, we obtain the following matrix(9)

Next, the classification of the prioritized genes can be done by diagonalization of the matrix C in Equation (9) by using the hierarchical clustering method. Furthermore, after matrix diagonalization, suppose the genes are divided into groups *G*_1_, *G*_2 _···,*G_l_*, and the adjacent nodes are divided into , then it is promising to analyze the subtypes of the phenotype *p *based on such divisions. And the resulted sub-groups of disease genes are likely to be related to the functional units of disease subtypes.

Finally, we statistically analyze the subgroups of genes to evaluate whether they are separable in terms of network topology. We calculate the Mean-Hitting-Time between pairs of predicted disease-causing genes, either within the same subgroup or between different subgroups, to assess the topological similarity. The Fisher's exact test [28] is employed to access whether gene pairs within the same subgroup are more topologically similar than gene pairs in separate subgroups.

### A case study on Breast cancer subtype detection

Breast cancer is known to be a carcinoma with highly heterogeneous [21] and its heterogeneity is more complicate than the results suggested by histopathological analysis alone [29], so it became necessary to find more molecular evidence to distinguish Breast cancer subtypes. Therefore, we take "Breast cancer" as a typical case to evaluate the performance of CIPHER-HIT for detection of disease subtypes.

**Figure 4:**
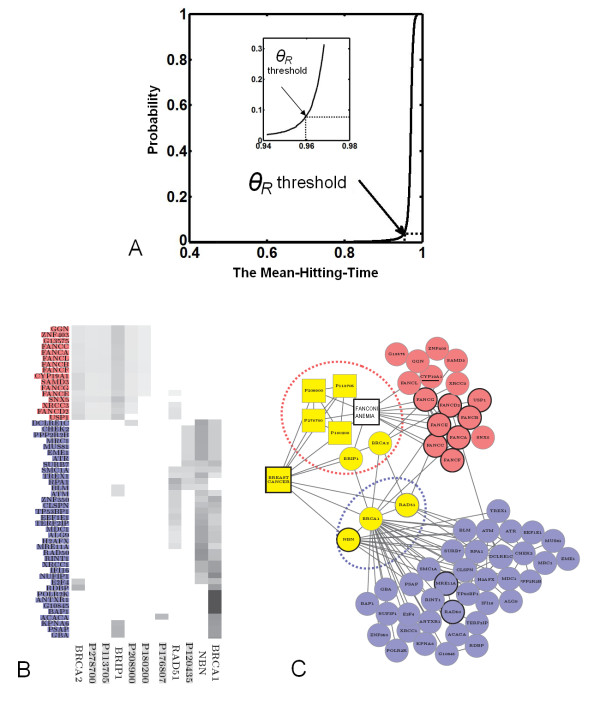
**Two subtypes of Breast cancer detected by CIPHER-HIT**. **(A) **The empirical distribution function of MHT(*g*,{*p*}) where *p *denotes the BREAST CANCER and *g *denotes all genes on the network. The *θ_R _*threshold = 0.96 at the critical point is selected in the Breast Cancer case. **(B) **The rows represent the similar phenotypes and disease-genes associated with Breast cancer and the columns represent the prioritized genes. The grey color indicates the closeness between an adjacent node and a prioritized node measured by the conditional Mean-Hitting-Time. Therefore the prioritized nodes are divided into two clusters in which the gene names of the nodes are displayed by red and blue respectively. **(C) **The yellow squares are the phenotypes with high similarity to Breast cancer and the yellow circles are the disease-genes associated with Breast cancer. For a better illustration, we left out two phenotypes (P120435 and P176807) in **(B) **with no connections to other nodes in the selected network. The blue and red circles denote two groups of prioritized genes by CIPHER-HIT. The module related to FANCONI ANEMIA locates in the cluster colored red and we added such a phenotype FANCONI ANEMIA in the graph. The protein complex RAD50/MRE11A/NBN locates in the cluster colored blue.

As shown in Figure 4A, the credible disease genes for Breast cancer predicted by CIPHER-HIT were filtered by the critical point of threshold *θ_R _*= 0.96 and resulted in a total of 155 credibly prioritized genes. Interestingly, by classification of the adjacent vectors described above, we found that it is worthwhile to note that 53 of the prioritized genes of Breast cancer can be divided into two groups (Figure 4B and 4C). The group containing the members of the Fanconi anemia gene family are tightly connected to the phenotypes FANCONI ANEMIA (OMIM ID: 227650), ATAXIA TELANGIECTASIA (OMIM ID: 208900), BREAST CANCER 1 GENE (OMIM ID: 113705), XERODERMA PIGMENTOSUM (OMIM ID: 278700) and the disease gene BRCA2. Another group is tightly related to the disease genes BRIP1, BRCA1, NBN and RAD51. BRCA1 is shared by both groups. In addition, the adjacent nodes of Breast cancer are divided into two parts, each of which leads to a sub-group of genes representing a subtype of Breast cancer. The two subtypes with genes obtained by the predictions of CIPHER-HIT not only have significant difference in topological features by Fisher's exact test (P < 0.0001 for both subtypes, see Table 1), but also yield agreements with the evidence reported by recent studies [25,26,30-34]. For example, the genes RAD50 and MRE11A in one of the predicted sub-groups are reported to form a protein complex related to Breast cancer [30]. Moreover, genes in the other predicted sub-group consist of FANCA, FANCB, FANCC, FANCD2, FANCE, FANCF and FANCG, which belong to the Fanconi anemia gene family, have been shown to be risk breast cancer susceptibility genes and contribute significantly to breast cancer predisposition [25,26]. The importance of genes involving in this subtype of Breast cancer is also supported by recent studies. For example, the polymorphisms of CYP19A1 (the aromatase gene) are closely related to the status and expression levels of estrogen receptor (ER) [31-33], HER2/neu [34] as well as progesterone [35]. Therefore, we suggest that the subtypes predicted by our method may serve as important genetic determinants that can influence the development of the well-known subtypes of breast cancer such as ER positive/negative, HER2 positive/negative, or progesterone receptor positive/negative [36,37].

**Table 1:** Statistical measures for the predicted two subtypes of Breast cancer*.

Disease subtypes (Disease subgroup)	Number of gene pairs with high topological similarity MHT(*g*, *g*') <*θ_R_*)	Number of gene pairs with low topological similarity MHT(*g*, *g*') >*θ_R_*)	P value#
Within subgroup 1	56	64	P_1_<0.0001
Within subgroup 2	333	570	P_2_<0.0001
Between subgroups 1 and 2	128	480	

*: We assess the modularity level of the predicted disease subtypes by comparing topological similarity of gene pairs within each subgroup to gene pairs between the two subgroups.

#: The P value of disease subgroup 1 (P_1_) and the P value of disease subgroup 2 (P_2_) are calculated using the Fisher's Exact Test.

Thus, the case study of Breast cancer shown in Figure 4 provides evidence that the connectivity features of the phenotype-gene heterogeneous network can be used to distinguish the molecular bases related to different disease subtypes and lead to novel findings. And the CIPHER-HIT method could serve as an important complementarity to current approaches for identification of cancer subtypes. If the prioritized genes of a queried phenotype are further divided into sub-groups which are related to subtypes of the disease, then we call each sub-group of genes as the susceptible modules of disease subtypes.

From the above example, it can be seen that the polymorphism of the cancer is related to a group of genes, instead of a single gene. We propose to characterize the subtypes of a disease by distinguishing the associated gene groups. If the adjacent nodes of a given phenotype exhibit a genetic or phenotypic difference, namely the prioritized genes can be divided into several sub-groups according to their relations to the adjacent nodes, it is likely to reveal subtypes according to a sub-division. Our work demonstrates that the disease subtype analysis can be carried out in the network context and benefit from the integration of phenotype and gene heterogeneous information. We also show that the modularity-based method, CIPHER-HIT, is a promising way to discover the subtype-associated genes based on the heterogeneous network structure. Based on the prioritization information on the gene sets, the results will allow for further clinical and experimental researches.

For the limitations of the present work, the CIPHER-HIT method currently only restricts on the genetic level, makes use of relatively simple data resources, and does not consider the quantitative analysis for gene expressions. As one of the future research directions, more efforts are still need to be made to evaluate the performance our method on different data, especially include quantitative information such as microarray and proteomics data for discovering disease mechanism in the gene expression level or protein level. An extension of our method to the systematic identification of disease subtypes also needs to be developed. Moreover, we believe that the method can also be easily generalized to enable the credible prediction of drug targets and detect the pleiotropic effects of drugs in our drugCIPHER framework [38] if we combine drug targets information into the phenotype-gene heterogeneous network.

## Conclusions

In summary, in this work, we introduce a concept of modularity level and propose a CIPHER-HIT method to use the Mean-Hitting-Time to measure global closeness between nodes of the heterogeneous network that consists of both genes and phenotypes. This measure has solid mathematics foundations and is easy to calculate. Based on this measure, we proposed a method to select high confident neighbors of a phenotype and detect gene modules that are highly connected to these high confident neighbors. Therefore the modularity of prioritized genes can be revealed, which may provide more mechanistic insights to the phenotype-genotype association. We also demonstrate that the performance of disease gene predictions is improved significantly by combining the modularity measure into the network inference, suggesting modularity is one of key features for network-based credible prioritization of candidate disease genes. Moreover, by detecting the sub-modules in the heterogeneous network, we revealed the potentially genetic and phenotypic properties of cancer subtypes. We believe this method can also be explored to predict biomarkers associated with disease subtypes in the gene expression and protein levels, as well as detect the pleiotropic drug actions in the future.

## Materials and methods

### Dataset and the heterogeneous network

We used the following three data sets to form the three parts, namely the phenotype-phenotype similarity network, protein-protein interaction network and gene-disease association network, of the phenotype-gene heterogeneous network based on which the prediction was carried out.

• The Human Protein Reference Database (HPRD) [39] was adopted to construct the protein-protein interaction network. The largest component of the HPRD protein-protein interaction network contains 34364 edges and 8503 vertices.

• The phenotype similarity came from the results calculated by van Driel *et al. *[17]. The phenotype similarity network contains 5080 phenotypes.

• The associations between the phenotypes and genes were from the OMIM (Online Mendelian Inheritance in Man, http://www.ncbi.nlm.nih.gov/omim) records as described in precious studies [2,4]. The edge weights of this phenotype-gene sub-network will be defined in Equation (10).

The heterogeneous network was described by the weight matrix. We constructed it by merging the weight matrices of the sub-networks into one matrix. Let *W_G _*denote the weight matrix of the HPRD network. For any two genes *g*_1 _and *g*_2_, if there was a corresponding protein-protein interaction recorded in the HPRD database, then *W_G_*(*g*_1_, *g*_2_) = 1, otherwise *W_G_*(*g*_1_, *g*_2_) = 0.

The phenotype similarities were used as the description of the diseases relations. The same data as previous works [2,4] were used, where the phenotype similarity data were calculated by van Driel *et al. *[17]. Since the high similarities were only present between parts of phenotype pairs, we set a threshold to filter out very low similarity values. Let *W_p _*denote the weight matrix of the phenotype-phenotype similarity network. If the similarity value between two phenotypes *p*_1 _and *p*_2 _was larger than the threshold 0.4, then the weight *W_p_*(*p*_1_, *p*_2_) took this similarity value, otherwise *W_p_*(*p*_1_, *p*_2_) = 0.

The phenotype-gene associations were taken from the same data set as [2,4]. If there was an association between phenotype *p *And gene *g*, then we specified the weight of the corresponding edge as(10)

by which we can achieve that for each pair of associated gene and phenotype (*g*, *p*), the average possibility of "walking" onto a different sub-network at the point *g *and *p *in the random walk process will equal 0.5.

Thus, the weight matrix of the heterogeneous network was constructed as(11)

where  refers to the transpose of *W_A_*.

We defined the random walk according to the weight matrix described as Equation (11) and carried out the network inference on it.

### The Mean-Hitting-Time and conditional Mean-Hitting-Time in CIPHER-HIT

In the previous random walk with restart method [3,4], the stationary distribution is used to define closeness between two nodes on a network. Here we define the topological properties on the phenotype-gene heterogeneous network in the same mathematical background using the Mean-Hitting-Time of the random walk. This definition is more suitable in solving the problem of both disease-causing gene inference and disease subtype inference, because by adopting this measure, we no longer have to choose the priori parameter required in the former method (which was always assumed to be arbitrary), and this measure leads us to a natural way of discovering modularity characteristics on the heterogeneous network. The math formula expressions below are mainly adopted from [40,41].

The random walk on the heterogeneous network was constructed by specifying its transition probability matrix P based on the weighted matrix W in Equation (11).(12)

The Mean-Hitting-Time from other nodes to a given node *p *could be obtained by solving the following Equation (13)(13)

where *I *refers to the identity matrix, and *x*(*v*) refers to the *v*th component of vector *x*.

The non-negative minimum solution  gave the Mean-Hitting-Time from all other nodes, both the gene-nodes and phenotype-nodes, to the given phenotype-node *p*. Furthermore, the conditional Mean-Hitting-Time  could be computed by solving(14)

where ℙ*_v_*(*τ_p _*<*τ_B_*), termed as the harmonic potential in the Markov Process theory, is the probability that a random walk starting from *v *reached *p *before *B*. The harmonic potential could also be obtained from the minimum non-negative solution of(15)

The theoretical proof of Equations (13), (14), and (15) is referred to [40,41].

## Authors' contributions

SL directed the research and discovered the relationship between the computational results and the biological evidence. XY and SL designed the whole methodology. XY and HH implemented the algorithm and the computation framework. YL provided constructive suggestions on this work. All the authors have read and agreed to the manuscript.
